# Digital Health Literacy in Adults With Low Reading and Writing Skills Living in Germany: Mixed Methods Study

**DOI:** 10.2196/65345

**Published:** 2025-05-22

**Authors:** Saskia Muellmann, Rebekka Wiersing, Hajo Zeeb, Tilman Brand

**Affiliations:** 1 Leibniz Institute for Prevention Research and Epidemiology - BIPS, Achterstraße 30, Bremen, 28359, Germany, 49 42121856916; ^2^Leibniz ScienceCampus Digital Public Health, Bremen, Germany; 3 Leibniz Living Lab, Bremen, Germany; 4 Faculty of Human and Health Sciences, University of Bremen, Bremen, Germany

**Keywords:** digital health literacy, digital health technologies, digital health, digital divide, mobile phone, adults, mixed methods study

## Abstract

**Background:**

Digital health literacy is a key factor in enabling users to navigate in an increasingly digitalized health care system. Low levels of digital health literacy are associated with higher age, low education, and income, as well as low functional health literacy. Around 6.2 million adults living in Germany have low reading and writing skills. Due to their low literacy, this group is often underrepresented in research studies and therefore little is known about their digital health literacy and use of digital health tools.

**Objective:**

The objectives of this study were to assess digital health literacy in adults with low reading and writing skills and to explore which digital health tools they use in daily life.

**Methods:**

An interviewer-administered survey and focus groups were conducted with adult residents of Bremen, Germany, who were aged 18‐64 years and had low reading and writing skills. In addition, a stakeholder workshop was held to derive recommendations on how digital health literacy could be improved. The survey questionnaire included 21 items addressing the use of digital health technologies and digital health literacy (eHealth Literacy Scale). Focus group participants completed several tasks on web-based health information and then discussed their experiences. Survey data were analyzed using descriptive statistics and linear regression. Qualitative content analysis was applied to analyze the focus group data and the written documentation of the stakeholder workshop.

**Results:**

Survey participants (n=96) were on average 43 (SD 10.7) years old, 72% (69/96) were female, and 92% (88/96) were not born in Germany. Participants reported mainly using information-related digital health technologies such as health apps (40/96, 42%), health websites (30/96, 31%), or activity trackers (27/96, 28%). The mean digital health literacy score was 22 (SD 8) points, with 35% (34/96) of participants classified as having a low digital health literacy (score between 8-19/40 points). Digital health technology use was associated with higher digital health literacy. For participants in the 5 focus groups (total n=39; mean age 43, SD 12.6 years; n=34, 87% female), limited technical skills and language problems were the most important challenges. Furthermore, focus group participants reported that they favor videos when searching for web-based health information and prefer to seek support from family members or local organizations for health issues. Stakeholders (n=15) recommended that health websites should be available in multiple languages, contain simple and easy-to-read language, and use images, symbols, and videos.

**Conclusions:**

While adults with low reading and writing skills use digital health technologies, many find it challenging to search for health information on the internet due to lacking technical skills and language problems. To ensure that adults with low reading and writing skills are not further left behind, future research should focus on developing tailored interventions to promote digital health literacy.

## Introduction

The digitalization of health care services, such as video consultations or prescribed digital health applications, is accompanied by a shift of prevention and health promotion activities and interventions to the digital environment (eg, wearables and online sport courses) [1,2]. Health information from the internet is easily and instantly available [3]. However, the large amount of health information on the internet is often overwhelming for users, and it is particularly difficult to use the information found to make health decisions [4]. The level of digital health literacy is a key factor in enabling users to navigate in an increasingly digitalized health care system. According to one well-known definition, digital health literacy describes the ability to seek, find, understand, evaluate, and apply health information from digital sources to one’s own health problem [5]. In addition, interaction with health apps (eg, for tracking physical activity) or interactions on social media platforms, including creating own content, are also included under current concepts of digital health literacy [6,7]. Hence, digital health literacy combines various subskills such as computer literacy (ie, use of computers and digital devices), information and media literacy (ie, critical use of information and media), science literacy (ie, knowledge and understanding of scientific principles, concepts, and processes), traditional literacy (ie, reading and writing skills), and health literacy [1]. Studies show that digital health literacy is unevenly distributed among population groups and influenced by sociodemographic factors such as age, education, income, and ethnicity. Older adults and persons with low levels of education and income have been found to have lower digital health literacy than their counterparts [6,8,9], whereas ethnicity was not linked with digital health literacy in the systematic review and meta-analysis by Estrela and colleagues [6]. One study found that being a Spanish-speaker in the United States was not associated with digital health literacy [10]. However, other studies found a lower level of digital health literacy among Arabic native speakers in Sweden [11] and non-English versus English-speaking parents of children with special health care needs in the United States [12]. The second Health Literacy Survey Germany did not find an association of digital health literacy with migration background per se, however, that survey showed an association of low functional health literacy, used as a proxy for general literacy (reading and writing skills and numeracy), with low digital health literacy [7,13]. This is important as an estimated 12% of the adult German population (ie, 6.2 million adults) have low reading and writing skills, meaning that they can only read and write simple sentences in German [14]. Around half of the participants with low reading and writing skills reported to have another native language than German. In addition, adults with low reading and writing skills are less likely to write emails or search for health-related information on the internet compared with adults with sufficient reading and writing skills [14]. Even though several studies have already investigated digital health literacy of the general population living in Germany [4,7,15] and various subgroups such as students [16], school children [17], teachers [18], physicians [19], or community inhabitants [20], very little is known about the digital health literacy of adults with low reading and writing skills and their use of digital tools for health purposes. Therefore, the main objectives of this study were (1) to assess the digital health literacy in adults with low reading and writing skills and (2) to explore which digital tools they use in daily life.

## Methods

### Study Design

This is an exploratory study using a mixed methods design consisting of a survey, focus group discussions, and a stakeholder workshop. The study report adheres to the Strengthening the Reporting of Observational Studies in Epidemiology (STROBE) [21] (Checklist 1) and Consolidated Criteria for Reporting Qualitative Research (COREQ) [22] (Checklist 2) guidelines.

### Participants

Inclusion criteria for the survey and focus group discussions were (1) age between 18 and 64 years; (2) living in Bremen, Germany; and (3) low reading and writing skills in German (ie, able to read and write simple sentences in German only; alpha level <4). Eligible participants could be native German speakers, adults who could read and write in their native language but not in German, or adults who could not read and write either in their native language or German.

### Procedure

We aimed to conduct 70 survey interviews and 5 focus groups with 6-8 participants each, striving for a balanced ratio of participants with different literacy levels (ie, native German speakers, good literacy in native language but not in German, and low literacy in either native language and German). Survey and focus group participants were recruited via a snowball system. Health mediators employed at the research institution for the project duration recruited participants via their local community networks (eg, mothers’ centers and integration courses). Flyers were displayed in various locations (eg, mothers’ centers and meeting centers for citizens) and distributed during events organized by our community office (ie, Leibniz Living Lab) or cooperating city district initiatives. In addition, the project was presented in German language courses and integration courses, and eligible participants were approached personally via the health mediators and their local networks. The health mediators had a native language other than German (eg, Russian or Turkish) but were fluent German speakers and, due to their main occupation, were well-connected in the community and with the people who had immigrated to Germany living there. Participants were also recruited from German language courses provided by adult education centers. The language courses aimed to teach reading and writing skills to native German speakers and people who have immigrated to Germany and could understand and speak German.

All participants recruited by the health mediators were asked to complete a 9-item test to determine their literacy level [23]. Participants recruited from adult education centers did not have to complete this test as their literacy level had already been assessed for their language course attendance.

### Survey Questionnaire

The structured interviewer-administered survey took approximately 30 minutes to complete and included 21 items covering three topic areas: (1) use and acceptance of digital health technologies (5 items), (2) digital health literacy (8 items), and (3) sociodemographic characteristics (8 items). The items were self-developed or adapted from existing surveys or instruments. The German version of the questionnaire can be found in Multimedia Appendix 1.

Use and acceptance of digital health technologies were assessed based on purposes of digital devices, use of digital devices in health context, use of digital health technologies (ie, list with 12 digital health technologies), perceived advantages, and disadvantages [24,25].

Digital health literacy was measured using the German version of the eHealth Literacy Scale (eHEALS) [26]. Each item was rated on a 5-point Likert scale ranging from strongly disagree to strongly agree.

Sociodemographic characteristics included age, sex, country of birth, spoken language at home, self-assessed speaking, reading, and writing skills in native language, highest educational degree, and employment status. Items for age, sex, country of birth, spoken language at home, highest educational degree, and employment status were adapted from the “German Health Update” 2019/2020 study [27]. The item for self-assessed reading and writing skills in native language was self-developed.

The interviewer-administered questionnaire was filled out by a health mediator or a member of the research team during a personal interview conducted in our community office (ie, Leibniz Living Lab), mother centers, or adult education centers. Based on the participant’s preference, the interview was conducted in German, English, Russian, Arabic, Turkish, Macedonian, Twi, or Tamil.

### Focus Group Protocol

The approximately 90-minute long focus groups were structured into four parts: (1) welcome and introduction, (2) task course, (3) overarching discussion, and (4) summary and farewell.

During the welcome and introduction, participants received the study information in verbal and written form, gave informed consent to participate, and filled out a short questionnaire on sociodemographic information (ie, age, sex, and employment status). During the focus group, participants were presented with 4 tasks which were developed on the basis of the Digital Health Literacy Instrument (DHLI) by van der Vaart and Drossaert [28]. For each of the 7 DHLI dimensions (ie, operational skills, navigation skills, information searching, evaluating reliability, determining relevance, adding content, and protecting privacy), a performance-based item was developed that included the application of a particular skill in a fictional situation [28]. We adapted the proposed items for our context and target group, focusing on the dimensions of operational skills, navigation skills, information searching, evaluating reliability, and determining relevance (Textbox 1). The tasks were not intended to measure participants’ digital health literacy. All tasks were formulated in simple language and were read out to the participants. In addition, pictures and symbols were used to describe the content of the tasks. Participants solved tasks individually on their smartphones or using a laptop provided by the study team. If necessary, participants were supported by the study team while completing the tasks. After each task, a group discussion on encountered difficulties and strategies to overcome difficulties was held (ie, what experiences did you have while working on the task? What was difficult for you? What did you do if you did not make any progress in the search?). In addition, the study team described a possible solution for each task. In the overarching discussion, participants discussed which digital devices they use, which formats (eg, videos) they prefer, and how they use web-based services for health-related purposes. At the end of the focus group, the study team provided a summary and gave out a handout to the participants with solutions to the tasks. Focus group discussions were conducted in German at our community office (ie, Leibniz Living Lab), mother centers, or adult education centers, and were moderated by at least 3 members of the study team. Focus group discussions were audio-recorded and transcribed verbatim. The focus group protocol is available from the authors upon request.

Textbox 1. **Tasks for focus group discussion.**

- Please open the internet on your laptop or smartphone. Please close the internet. Please open the website www.bremen.de (website with information and facts about the city of Bremen). Please open a search engine such as Google search (Digital Health Literacy Instrument [DHLI] dimensions: operational skills, navigation skills).
- Please search the internet for a general physician in your neighborhood. Note the telephone number to make an appointment (DHLI dimensions: information searching).
- Please open the website www.gesundheitsinformation.de (website for independent and evidence-based health information). When does a child with fever need to see a physician? (DHLI dimensions: information searching, determining relevance)
- Please search the internet. When do you need to see a physician if you have severe back pain? (DHLI dimensions: information searching, evaluating reliability, determining relevance)

### Stakeholder Workshop

The main project results were presented and discussed with local stakeholders in a workshop conducted in our community office (ie, Leibniz Living Lab) using the world café method [29]. Stakeholders were recruited via a snowball system. Thus, stakeholders from civil and public services, local public authorities, and community social service providers or groups that work with adults with low reading and writing skills and are located in Bremen were asked to participate in the workshop. After presenting project results (ie, survey and focus group discussions), the stakeholders split into three groups (approximately 5 stakeholders per group) and discussed the following questions at group tables for 15 minutes each: (1) which competencies are needed by adults with low reading and writing skills to use digital health technologies, (2) how should digital health technologies be designed for adults with low reading and writing skills, and (3) which and how stakeholders should be involved in making digital health technologies for adults with low reading and writing skills accessible. After 15 minutes, the next group went to the table until all 3 groups had discussed all the questions. Each group table was moderated by 1 researcher (SM, RW, or TB) and 1‐2 health mediators. The results of the world café were documented on meta-plan paper. In addition, discussion notes for each group and photo documentation were completed. All participants received a copy of the discussion notes and world café results.

### Data Analysis

Filled-out survey questionnaires were entered independently into the software LimeSurvey by 2 members of the study team (SM and Finola Goepfert; refer to Acknowledgments). The data were then imported into IBM-SPSS 24 (IBM Corp), cleaned, and prepared for statistical analysis. The responses to all survey items were analyzed descriptively using absolute and relative frequencies or means and SDs. Linear ordinary least square regression models were computed to investigate the association between digital health literacy and participant characteristics including age, sex, formal education, language skills, and use of digital health technologies. Based on the distribution of scores, formal education was categorized into 3 groups (low: less than 9 years of schooling, no formal degree; medium: 9‐10 years of schooling, lower secondary education; and high: 12‐13 years of schooling, university entrance qualification), employment status into 5 groups (full-time employed, part-time employed, care work, language course, and other), and use of digital health technologies into 2 groups (no use vs use of at least 1 digital health technology). Participants were grouped into three categories of language skills based on language spoken at home and self-assessed reading and writing skills in native language: (1) native German speakers, (2) good literacy in native language but not in German, and (3) low literacy in both native language and German. For digital health literacy, two variables were computed: (1) total eHEALS score, ranging from 8 (lowest digital health literacy) to 40 points (highest digital health literacy) and (2) a 3-category variable based on the study by De Santis and colleagues [24] (low: 8‐19 points, medium: 20‐29 points, and high: 30‐40 points).

Qualitative content analysis [30] following a deductive-inductive approach was applied to analyze the focus group data. First, focus group transcripts were imported into MAXQDA 2020 (VERBI–Software Consult Sozialforschung GmbH) and relevant text units were coded into overarching categories and subcategories. Categories and subcategories were derived from the focus group protocol (deductive coding). New categories and subcategories were created if text units did not fit into any of the pre-existing categories or subcategories (inductive coding). Second, data were grouped and summarized into themes using Microsoft Excel. Coding, grouping, and summarizing of the data was performed by one researcher (SM) and checked by a second researcher (Meret Lakeberg; refer to Acknowledgments). Any discrepancies in the coding or reduction process were discussed until a consensus between researchers was reached. Illustrative quotes were selected from the existing material as anchor examples and translated into English. All translations were checked by a native English speaker.

As this is an exploratory study, no sample size calculation was conducted.

### Ethical Considerations

This study was approved by the Ethics Committee of the University of Bremen, Germany, on December 6, 2022 (reference 2022‐29), in accordance with the ethical guidelines for human participant research and the Declaration of Helsinki. All participants received verbal and written information about the study and gave informed consent (Multimedia Appendix 2) for their data to be used. All data were anonymized to ensure privacy and were securely stored on a password-protected server at the research institution accessible only to the research team. Participation in the survey and focus group discussions was voluntary, could be terminated at any time, and was reimbursed with €15 (US $17). No identifiable participant information has been included in the manuscript or supplementary materials.

## Results

### Survey

A total of 108 interviews were conducted between December 2022 and March 2023. Mean interview duration was 29 (range 6-75) minutes. Of the total, 12 interviews were excluded based on the following reasons: participants aged 65 years and above (n=5) or not meeting criteria for low literacy (n=7). The remaining 96 interviews were included in the analysis. Of these, around half were conducted in German (n=47, 49%), 13 in Russian (14%), 12 in Twi (13%), 8 in Arabic (8%), 6 in English (6%), and 4 in Turkish (4%). None of the interviews was conducted either in Macedonian or Tamil, respectively. For 6 interviews, no information on the conducted language is available. Interview participants were on average 43 (SD 10.7) years old, 72% (69/96) were female, 92% (88/96) were not born in Germany, 38% (36/96) reported a medium level of formal education, and 30% (29/96) attended a language course (Table 1). In total, 11 participants (12%) were German native speakers, 32 participants (33%) reported good literacy in their native language but not in German, and 53 participants (55%) had low literacy in both German and their native language. Mean digital health literacy score was 22.2 (SD 8) points ranging from 8 to 40 points. Digital health literacy was classified as low in 35% (n=34), medium in 44% (n=42), and high in 19% (n=18) of participants. In addition, 59 participants (62%) were digital health technology users.

Regression analysis found no associations between age, sex, and digital health literacy (Table 2). Digital health literacy was approximately 6 scale points lower among participants with low language skills in both their native language and German as compared with native speakers. Participants with a medium level of formal education reported a higher digital health literacy compared with those with no formal degree. Furthermore, the average digital health literacy score was approximately 10 points higher among digital health technology users compared with nonusers.

**Figure 1.**
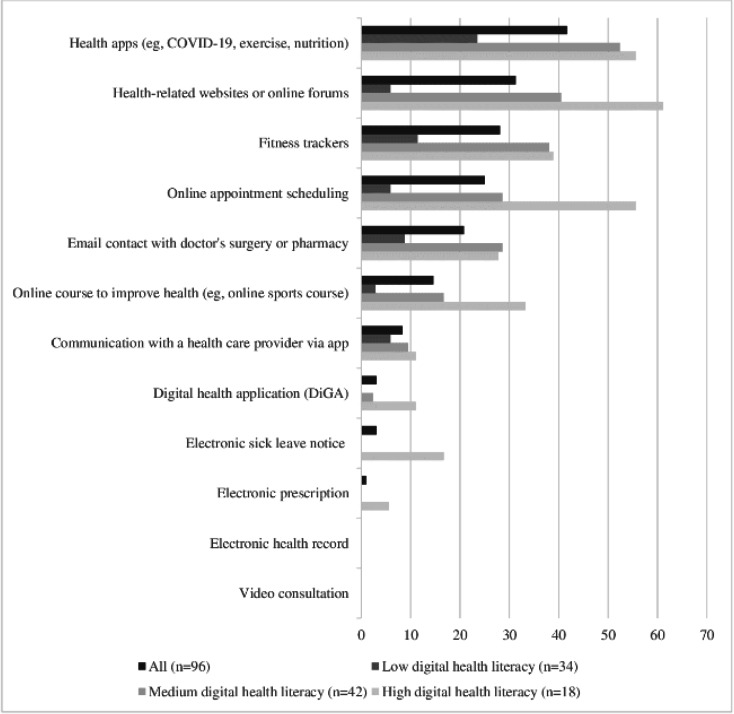
Percentages of adults who used different digital health technologies for health-related purposes, by level of digital health literacy (multiple answers allowed).

The 5 most commonly used health-related digital technologies were health apps (40/96, 42%), health-related websites or forums (30/96, 31%), fitness trackers (27/96, 28%), web-based appointment scheduling (24/96, 25%), and email contact with doctor’s surgery or pharmacy (20/96, 21%) (Figure 1). Health care–related digital technologies, such as prescribed digital health applications (3/96, 3%), electronic sick leave notice (3/96, 3%), or electronic prescription (1/96, 1%), were used by very few participants. None of the participants reported accessing their electronic health record or using video consultations. Participants with low digital health literacy used fewer digital health technologies than those with medium or high digital health literacy, and specifically none of the health care–related options (Figure 1). Results stratified by language skills showed higher usage of digital health technologies by native German speakers and adults with good native language literacy compared with participants with both native language and German literacy (Multimedia Appendix 3).

Most participants (n=74, 77%) used a smartphone or tablet to access health-related information (Table 3). The most common reasons for using digital health technologies were time savings (n=44, 46%), access at anytime from anywhere (n=42, 44%), motivation to stay healthy (n=39, 41%), and cost-effectiveness (n=38, 40%). Reasons for not using digital health technologies included preference for personal advice (n=41, 43%) or paper-based information (n=33, 34%), concerns about data security (n=20, 21%), mistrust in health information (n=19, 20%), technical problems (n=9, 9%) and lack of a suitable device (n=6, 6%).

**Table 1.** Participant characteristics.

Characteristic		Survey (n=96)	Focus groups (n=39)^a^
Age (years), mean (SD)		43.1 (10.7)	43.1 (12.6)
Sex, n (%)			
	Female	69 (72)	34 (87)
	Male	27 (28)	5 (13)
Country of birth, n (%)			
	Germany	8 (8)	—^b^
	Other than Germany	88 (92)	—
Formal education^c^, n (%)			
	Low	37 (39)	—
	Medium	36 (38)	—
	High	22 (23)	—
	Unknown	1 (1)	—
Employment status, n (%)			
	Full-time employed	15 (16)	3 (8)
	Part-time employed	10 (10)	6 (15)
	Care work	23 (24)	8 (21)
	Language course	29 (30)	18 (46)
	Other (eg, job seeking, unable to work)	17 (18)	4 (10)
	Unknown	2 (2)	0 (0)
Language skills, n (%)			
	Native German speakers	11 (12)	—
	Good literacy in native language but not in German	32 (33)	—
	Low literacy in both native language and German	53 (55)	—
Digital health literacy			
	Mean score (SD)	22.2 (8)	—
	Low (8‐19 points), n (%)	34 (35)	—
	Medium (20‐29 points), n (%)	42 (44)	—
	High (30‐40 points), n (%)	18 (19)	—
	Unknown, n (%)	2 (2)	—
Use of digital health technologies, n (%)			
	User	59 (62)	—
	Nonuser	37 (38)	—

a Only age, sex, and employment status were assessed.

b Not assessed.

c Low: less than 9 years of schooling, no formal degree; medium: 9‐10 years of schooling, lower secondary education; high: 12‐13 years of schooling, university entrance qualification.

**Table 2.** Bivariate associations between digital health literacy and participant characteristics (linear regression models).

Participant characteristics		Unstandardized linear regression coefficient (95% CI)
Age		−0.05 (−0.21 to 0.10)
Sex		
	Male	Reference
	Female	−0.48 (−4.12 to 3.14)
Language skills		
	Native German speakers	Reference
	Good literacy in native language but not in German	1.59 (−6.69 to 3.51)
	Low literacy in both native language and German	−6.10 (−11.4 to −0.73)
Formal education		
	Low	Reference
	Medium	5.85 (2.30 to 9.40)
	High	1.77 (−2.28 to 5.81)
Use of digital health technologies		
	Nonuser	Reference
	User	9.94 (7.24 to 12.66)

**Table 3.** Use of digital devices in health context and reasons for use and non-use of digital health technologies.

Digital device use in health context and reasons		Value, n (%)
Use of digital devices in health context^a^		
	Computer or laptop	19 (20)
	Smartphone or tablet	74 (77)
	Activity tracker or smartwatch	19 (20)
	Gaming console	7 (7)
Reasons for use of digital health technologies^a^		
	It saves time	44 (46)
	I have access at any time and from anywhere	42 (44)
	It is cost-effective	38 (40)
	I want to stay healthy	39 (41)
	I am interested in digital health technologies	28 (29)
	I trust the information	25 (26)
	I otherwise have difficulties accessing healthcare services	14 (15)
	Other reasons	5 (5)
Reasons for nonuse of digital health technologies^a^		
	I prefer information on paper	33 (34)
	I prefer personal advice	41 (43)
	I have poor internet access	10 (10)
	I have technical problems	9 (9)
	I do not have a suitable device	6 (6)
	I do not know if my data are secure	20 (21)
	I do not trust the information	19 (20)
	I do not have interest in such offers	7 (7)
	Other reasons	20 (21)

a Multiple responses allowed.

### Focus Groups

A total of 5 focus groups (total n=39 participants) were conducted between April and June 2023. Participants were on average 43 (SD 12.6) years old, 87% (34/39) participants were female, and 46% (18/39) attended a language course (Table 1).

Anchor examples for the reported difficulties and strategies to overcome difficulties can be found in Table 4. Due to technical difficulties in using digital devices (eg, computer) or the use of cookies on websites, searching for health information on the internet was difficult for participants. Most focus group participants reported language problems in comprehending technical and foreign words on health information websites and reading and writing German. The lack of translation options (eg, calling family members or friends with better German skills or unstable internet connection for using translation services) while filling out German-language forms in medical practices was also reported as a difficulty. In addition, participants reported difficulty in using search engines to find important and relevant information on the internet. Some participants mentioned that they were unsure on how to use or search on the internet because they were afraid of ordering something.

Most focus group participants stated that they seek advice from family members, friends, acquaintances, or local organizations when they encounter difficulties while searching for health information on the internet. Participants also tried to find a solution themselves by combining word and image searches or using translation tools. Some participants relied on their own experience (eg, if a child has fever) or on previous knowledge and thus did not consider searching for health-related information on the internet. In general, most focus group participants stated that they preferred personal or telephone contact with health care providers (eg, to arrange doctor’s appointments or for acute medical concerns).

In the overarching discussion, focus group participants reported which digital devices, formats, languages, and digital health technologies they have used so far (Table 4). Participants used smartphones to search for information on the internet. Information was sought in German, in the native language, or in a combination of German and the native language. Participants who could not or could barely read and write in their native language sought information in German, whereas participants who could read and write well in their native language sought information in their native language or a combination of German and their native language. Participants mainly used videos or a combination of different formats such as video and text. They also reported searching for images or using voice assistants.

**Table 4.** Examples of difficulties in using digital health technologies and strategies to overcome difficulties reported in focus group discussions.

Difficulties and strategies		Anchor examples
Difficulties		
	Use of digital device	*If you can [work] with the computer, it’s not difficult. […] I can with a smartphone. But with a laptop or computer I can’t.* [ID02, task 1]
	Cookies	*For me, cookie[s] were always [difficult] to accept. It was very, very difficult for me.* [ID03, task 3]
	Reading, writing and understanding German	*For me, sometimes I have problem. Not all doctor or doctors can speak English, and my German [is] not so good. So sometimes when I look on the internet and sometimes my idea is: This doctor speaks English, or a little English? Or not? So sometimes is a bit difficult for me when I take a doctor from the internet.* [ID05, task 2] *Internet not difficult, but sometimes we don’t understand German. That’s why [it’s] a bit difficult for me. [...]* [ID04, task 2]
	Translation	*And one problem is when first visiting, they give all the papers [to fill out]. And all is in German. And if someone can’t understand everything, yes, once I [asked] the nurse, because I can’t understand everything, my German is not good. But I can’t help you, you have to do it on your own. And they don’t have internet to translate. [...]* [ID05, task 2]
	Technical terms and foreign words	*It wasn’t difficult for me, but I don’t understand the words, [for example] physiotherapy. The words. I don’t know [if they] mean this or that, and I don’t understand all of them. There’s a lot of information, that’s the problem.* [ID05, task 4]
	Searching and finding health information	*It was a bit difficult this time. So you have to search and read at the same time. I also got a bit of help. [Interviewer: But what was more difficult?] So writing down this information. And to find it. [...]* [ID03, task 3]
	Uncertainty	*So I can open it, I can see everything. But I’m afraid to order something. [...] That’s why I don’t dare to do that. [...]* [ID02, task 1]
Strategies used to overcome difficulties		
	Seek support from family, friends, acquaintances, or local organizations	*It’s easy with me, but I’m a bit of a problem. I ask my colleague or my children, or. But now it’s not difficult for me.* [ID02, task 2] *[...] We can also enter, we have done a lot, because now Bremen is full of people from Ukraine and then you can enter directly into Google: Ukrainian-speaking doctors. Or we have another possibility and each of us, not just people from Ukraine, from Syria, from Persia, from Iran. We stick together, we share experiences. It’s an organization called Bras. And when you go [there], you get in touch. Give your own details, then at a doctor’s appointment, you get an interpreter.* [ID03, task 2]
	Try again	*We’ll try next day [laughs]. If I don’t make it today, I’ll try next day.* [ID02, task 2]
	Draw on own experiences or previous knowledge	*In our home country our mother has a picture of fever, for fever goes down. That is a warm, a little warm, a little not warm water [Interviewer: Poultice?] Yes, every parent from Africa, for example Ghana, knows that if the child has a fever and you have no medication then you do that.* [ID05, task 3]
	Combine word and image search	*Some words I don’t understand well. I write on Google with German and then I see photos. Because I can’t write in my own language. I write German and then photo comes up and I see photo and understand what it means.* [ID02, task 1]
	Use translation function	*For me [it] is a bit difficult because I know many words in German - I don’t understand it. So, I have to copy and then translate in Google Translator and sometimes the rest of the words from German it gives me wrong in English or different idea.* [ID05, task 4]
	Arrange a doctor’s appointment by phone or in-person	*When I’m looking for a doctor, I prefer to call, also because sometimes they want to have it that way. Then I make an appointment and at home I have a calendar of when I can go and then I do it.* [ID02, task 2]
	Personal contact to medical practice	*You just go there and you have back pain or something. You don’t have to call or google it first. It’s much easier than going anywhere else.* [ID01, task 4]
Overarching discussion		
	Use of digital device	*It’s faster and easier. Computers, some people don’t know how to use computers, they can’t cope, so it’s easier with a smartphone.* [ID03, overarching discussion]
	Language	*I do it in German. Because I speak Arabic and Kurdish, but I can’t read and write because I didn’t go to school in my country, only in Germany. I do everything in German.* [ID01, overarching discussion] *And if there’s something I don’t understand, then I go to the dictionary or translation, so you can learn. If you always switch to your own language, you’ll never learn German.* [ID03, overarching discussion] *For health, I don’t go to German. It’s important and you have to understand everything. That’s why I just go to English.* [ID05, overarching discussion]
	Format	*Yes, video, because text is difficult for me a bit, because I don’t read well and it’s difficult to understand, but videos are easy and I always watch and have to understand (which teachings).* [ID04, overarching discussion] *It depends on what you need […]. Sometimes text is enough, sometimes video, sometimes like this.* [ID05, overarching discussion] *[...] If I don’t feel like writing, I speak into the microphone. Then I do that, then it works well, yes.* [ID02, task 2]
	Use of digital health technologies	*I usually go straight away. [Interviewer: And why directly to the doctor?] Yes, it’s easier. I call for an appointment and then I go.* [ID03, overarching discussion] *So I’ll check the Internet first. If that gives me the right answer. If not, I sometimes call my mother and ask her. If she has any ideas. If that doesn’t help, I go to the doctor.* [ID01, overarching discussion] *So I just search the Internet when I need offers for health. It’s much easier for me. [...] If I’m looking for a medicine, for example painkillers, ointment for back pain, they show it straight away and which pharmacies are close to me.* [ID03, overarching discussion]

### Stakeholder Workshop

In total, 15 stakeholders (n=8 from civil and public services, n=1 from local public authorities, and n=6 from community social service providers or groups) and 8 members of the study team (n=3 researchers and n=5 health mediators) took part in the 150-minute stakeholder workshop at our community office (ie, Leibniz Living Lab) in October 2023. Discussions highlighted the following: First, to be able to use digital health technologies, adults with low reading and writing skills need to be curious and open to learning and trying out new things. They also need to be patient, have a high tolerance for frustration, and be willing to ask for support. Second, basic (German) language comprehension and interpretation of figures and symbols are as necessary as the financial resources to purchase digital devices. Third, health information websites should contain little, simple, and easy-to-read language, be available in multiple languages, barrier-free (eg, by integrating a read-aloud function), and compatible with smartphones. In addition, they should have easy navigation with few “clicks” to the searched content, no time limits, and use of images, symbols, and videos. Fourth, health programs should be named rather than using nondescriptive project acronyms. Fifth, web-based health services should refer to local contacts or organizations (eg, in the city district) and instead of a chat function, persons with low reading and writing skills may benefit from a talking chatbot. Finally, stakeholders who should be involved are health professionals, health and educational institutions, physicians, health insurance companies, authorities, and stakeholders with ongoing health projects. To enable access to stakeholders not only digitally but also via analog channels, these should have fixed structures on site (eg, an office in the city district). Relevant stakeholders should be connected regularly, for instance, through the establishment of a working group. In addition, stakeholders should be educated and trained to pass on knowledge about web-based health services to adults with low reading and writing skills.

## Discussion

### Principal Results

This study assessed digital health literacy in adults with low reading and writing skills, explored which digital tools they use, and how digital health literacy can be improved from a stakeholder’s perspective. Most participants had a low to medium digital health literacy. Digital health literacy was particularly low among those with low reading and writing skills both in their native language and in German and among those without a formal schooling degree. Participants reported using mainly information-related digital health technologies such as health apps, health websites, or activity trackers. Use of digital health technologies was associated with higher digital health literacy. Language problems and lack of technical skills made it difficult for participants to search for web-based health information, so support for health-related questions is mainly sought from family members, friends, or health care providers (eg, physicians).

### Comparison With Previous Work

Adults with low reading and writing skills use digital health technologies, but less frequently compared with other population groups. For instance, compared with an online survey of adults insured at a statutory health insurance company in Germany [31], adults with low reading and writing skills reported lower usage of health websites (30/96, 31% vs 755/1678, 45%) and online appointment scheduling (24/96, 25% vs 1359/1678, 81%). In addition, similar to results reported by Schaeffer et al [7], use of digital health technologies in adults with low reading and writing skills is associated with higher levels of digital health literacy.

Compared with 2 cross-sectional telephone surveys of internet users living in Germany, the digital health literacy level was much lower in our study (mean eHEALS score of 22 points vs 31 points in survey 1 and 30 points in survey 2) [4,24]. This is also evident when comparing the eHEALS scores of the included studies in the systematic review by Estrela and colleagues [6] with our study. The included studies reported mean eHEALS scores ranging from 25 to 33 points. However, the authors stated that higher eHEALS scores were observed in studies targeting medical staff and that were conducted online or via phone. Furthermore, 2 more recently published systematic reviews and meta-analyses support these results [32,33]. In the reviews, mean eHEALS scores between 26 and 33 points [32] and between 20 and 29 points [33] were reported. Both reviews included studies with diverse population groups such as adolescents, university students, adults (ie, general adult population, army personnel, healthy adults, and adults with chronic diseases), or older adults. However, none of the included studies targeted adults with low reading and writing skills.

As several studies have shown, digital health literacy is unevenly distributed across populations with vulnerable groups such as older adults, persons with low levels of education and income reporting particularly low levels of digital health literacy [4,6-9,32]. More than one-third of our participants stated that they had no formal education. Low education and functional literacy appear to be cumulative resulting in a particularly low digital health literacy in adults with low reading and writing skills. Klinger and colleagues [13] come to similar conclusions; persons with low literacy skills and German proficiency have greater difficulties processing health information. This underlines the need for multilingual information in simple language and multimedia materials such as videos which was also supported by results from our focus group discussions and the stakeholder workshop. Best practices for researchers that should be considered when working with persons with limited literacy are, for instance, the use of graphics and visualizations, the consideration of cultural differences in the interpretation of graphics and phrases, the critical review of the wording of written materials, and the consideration of the digital (health) literacy of the target group [34]. However, even if reliable health information in various formats is available on the internet such as on the website for reliable and understandable information about health of the German Federal Ministry of Health [35], such information may not be accessible for persons with low and reading and writing skills due to lacking technical skills. Stakeholders responsible for community health projects and who are contacted for health purposes can improve (web-based) health information accessibility for adults with low reading and writing skills. However, to ensure that stakeholders can provide reliable information to adults with low reading and writing skills, they need sufficiently high levels of digital health literacy.

Our results are particularly important in the light of the ongoing shift of health and social services toward digital information exchange, contact provisions, and service inquiries. Given the lower digital competencies of persons with low reading and writing skills, more focus is needed on the risk of leaving less literate groups behind or reducing their options for interaction via their preferred, often nondigital pathways.

### Strengths and Limitations

To our knowledge, this is the first study that assessed digital health literacy and digital technology use of adults with low reading and writing skills in Germany. The recruitment strategy via health mediators and adult education centers was successful and more participants than initially planned took part in the survey. However, this study has several limitations. First, selection bias occurred as we were not able to recruit a sufficient number of German native speakers. In addition, more females than males participated in the survey and focus group discussions. Second, some participants had difficulty answering the survey questions, in particular the eHEALS items. For the eHEALS items, differences between the individual items were hardly recognizable for participants, even if the interview was conducted in their native language. Cutoff scores for the eHEALS sum score were built using the cutoffs proposed by De Santis and colleagues [24]. However, it should be noted that there are no uniformly used cutoff scores reported in the literature. Third, even though sufficient knowledge of German was required for participating in the focus groups, the discussions sometimes proceeded sluggishly due to language problems, and some participants may not have spoken as openly as they would have done if the focus group discussion had been done in their native language. Fourth, analysis of the qualitative data (ie, coding, grouping, and summarizing) was conducted by one researcher and checked by a second researcher. This ensures objectivity, but the gold standard would be to analyze the data or a part of the data by 2 researchers independently. However, this was not possible due to limited resources. Fifth, there are further methodological weaknesses (eg, no sample size calculation was conducted) inherent in the exploratory design of the study. This study aimed to gain first insights into the digital health technology use and digital health literacy of adults with low reading and writing skills in Germany. Based on the results, a methodologically more sophisticated study can be conducted in which, for instance, an intervention to promote digital health literacy in adults with low reading and writing skills is evaluated in a randomized controlled trial.

### Future Directions

The results of this study indicate a need for studies that develop and evaluate interventions to promote digital health literacy for adults with low reading and writing skills. In total, 1 scoping review and 1 meta-analysis investigated interventions to enhance digital health literacy [8,33], but these reviews focused on study populations of children, adolescents, older adults, or adults with diabetes, cardiovascular diseases, or other health conditions, and not adults with low reading and writing skills. Most of the included interventions focused on educational training to improve health-related knowledge and skills and were delivered in-person, via the internet (eg, app, website, and online platform), or through a combination of in-person and web-based sessions [8,33]. Our findings also suggest that interventions to improve digital health literacy in adults with low reading and writing skills should focus on improving technical skills and how to search for web-based health information (eg, where and how to find reliable health information on the internet). In addition, practical sessions should be included similar to the tasks used in our focus group discussions. Based on learnings from our study, interventions to improve digital health literacy should be delivered using a combination of in-person and web-based modalities (eg, videos). Furthermore, our research underscores the importance of considering the low digital health literacy level of adults with low reading and writing skills. In addition, 2 previous literature reviews pointed out that digital technologies so far have not considered digital health literacy levels of the target group [36,37] and, in particular, persons with low reading skills who may have different reading and design requirements and needs [36].

An additional future research direction is to further develop the tasks used in the focus group discussions to create a more objective tool for assessing digital health literacy. There are several instruments for measuring digital health literacy available [38]. Existing instruments are based on self-reports, and some instruments, such as the widely used eHEALS scale, only relate to searching for health information on the internet (Web 1.0) and do not include interactions on social platforms (Web 2.0) [28,38]. For the focus group discussions, some of van der Vaart and Drossaert’s performance-based items [28] were adapted to gain more in-depth knowledge on the challenges adults with low reading and writing skills face while searching for health-related information on the internet. The tasks gave valuable insights into the digital health literacy of our participants and can serve as a basis for further developing existing DHLI.

### Conclusions

This mixed methods study showed that while adults with low reading and writing use digital health technologies, many have low to medium digital health literacy and find it challenging to search for health information on the internet due to lack of technical skills and language problems. Tailored interventions to promote digital health literacy are needed to ensure that adults with low reading and writing skills are not further left behind.

## Supplementary material

10.2196/65345 Multimedia Appendix 1 German version of survey questionnaire.

10.2196/65345 Multimedia Appendix 2 Informed consent form.

10.2196/65345 Multimedia Appendix 3 Percentages of adults who used different digital health technologies for health-related purposes, by language skills.

10.2196/65345 Checklist 1 STROBE (Strengthening the Reporting of Observational Studies in Epidemiology).

10.2196/65345 Checklist 2 COREQ (Consolidated Criteria for Reporting Qualitative Research) checklist.

## Abbreviations

COREQ: Consolidated Criteria for Reporting Qualitative Research
DHLI: digital health literacy instrument
eHEALS: eHealth Literacy Scale
STROBE: Strengthening the Reporting of Observational Studies in Epidemiology
