# Supplementary material for: Digital Health Literacy in Adults With Low Reading and Writing Skills Living in Germany: Mixed Methods Study
Source: JMIR Hum Factors. 2025 May 22;12:e65345. doi: 10.2196/65345 (PMC12121537; doi:10.2196/65345)
Supplement: Multimedia Appendix 1 [file humanfactors-v12-e65345-s001.pdf]

Interviewer-ID: |__|__|

Fragebogen Forschungsprojekt Liter@te

Liebe Mediatorinnen,

Bitte beachtet beim Ausfüllen folgende Hinweise:

- Kennzeichnet die gewählten Antworten mit einem Kreuz: ☒
- Kreuzt nur eine Antwort an, es sei denn es wird ausdrücklich darauf hingewiesen, dass mehrere Antworten möglich sind.
- Wenn eine Antwort als Text aufgeschrieben werden soll, benutzt bitte die dafür vorgesehenen Linien: ______________
- Bitte überspringt eine Frage nur bei folgenden Hinweis: → *weiter mit Frage..*
- Wenn eine angekreuzte Antwort korrigiert werden soll, streicht diese bitte ganz durch und kreuzt die gewünschte Antwort an.
- Die Interviewerhinweise (in *kursiv*) bitte nicht vorlesen.
- Bitte nicht die Überschriften der Frageblöcke (Nutzung digitaler Geräte und digitale Gesundheitskompetenz, Alpha-Diagnostik Lesen, Soziodemographie) vorlesen.

Interviewsprache	○ 1	Deutsch
	○ 2	Englisch
	○ 3	Russisch
	○ 4	Arabisch
	○ 5	Twi
	○ 6	Türkisch
	○ 7	Mazedonisch
	○ 8	Tamil
Datum	_____._____.________<br>TT MM JJJJ	
Beginn des Interviews	______:______ Uhr	

Interviewerhinweis: Bitte ab hier die Zeit stoppen. Wird später für die Alpha-Kurzdiagnostik benötigt.

Ich werde während des Interviews die Zeit stoppen, damit ich weiß wie lange unser Interview gedauert hat.

Nutzung digitaler Geräte und digitale Gesundheitskompetenz

Zunächst möchte ich erfahren, welche digitalen Geräte und Gesundheits-angebote Sie nutzen und wie Sie mit digitalen Gesundheitsinformationen umgehen.

1 Welche der folgenden Funktionen am Computer, Laptop, Tablet oder Smartphone nutzen Sie?

Interviewerhinweis: Mehrfachantworten möglich

- ○ E-Mail
- ○ Kurznachricht (SMS oder Messenger)
- ○ Sprachnachricht
- ○ Videoanruf
- ○ Diktierfunktion
- ○ Vorlesefunktion
- ○ Sprachassistent (z.B. Alexa, Google Assistant, Siri)
- ○ Übersetzungs-App

2 Welche digitalen Geräte nutzen Sie im Zusammenhang mit Ihrer Gesundheit (z.B. um im Internet nach Gesundheitsinformationen zu suchen oder um Ihre Gesundheit zu verbessern)?

Interviewerhinweis: Mehrfachantworten möglich

- ○ Computer oder Laptop
- ○ Smartphone oder Tablet
- ○ Aktivitätstracker oder Smartwatch
- ○ Spielekonsole (z.B. Playstation)
- ○ Andere, welche? ________________________________
- ○ Gar keine → *Interviewerhinweis: weiter mit Frage 5*

3 **Welche der folgenden digitalen Gesundheitsangebote und Anwendungen haben Sie bereits genutzt?**

Interviewerhinweis: Mehrfachantworten möglich

- ○ E-Mail-Kontakt mit einer Arztpraxis oder Apotheke
- ○ Online-Terminvereinbarung (z.B. über eine Webseite oder ein Online-Portal zur Terminvereinbarung)
- ○ Kommunikation mit Gesundheitsdienstleister über App
- ○ Elektronische Arbeitsunfähigkeitsbescheinigung (AU)
- ○ Videosprechstunde
- ○ „App auf Rezept" bzw. Digitale Gesundheitsanwendungen (DiGA)
- ○ Elektronisches Rezept (eRezept)
- ○ Elektronische Patientenakte (ePA)
- ○ Webseiten oder Foren mit Gesundheitsinformationen
- ○ Online-Kurse zur Verbesserung der Gesundheit (z.B. Online-Sportkurs)
- ○ Gesundheits-Apps (z.B. COVID-19, Bewegung, Ernährung)
- ○ Bewegungsmesser (z.B. Fitnesstracker, Smartwatch)
- ○ Ich nutze keine digitalen Gesundheitsangebote und Anwendungen
  → *Interviewerhinweis: weiter mit Frage 5*

4 **Ich nutze digitale Gesundheitsangebote und Anwendungen, weil…**

Interviewerhinweis: Mehrfachantworten möglich;
anschließend weiter zu Frage 6

- ○ es mir Zeit spart
- ○ ich jederzeit und von überall Zugriff habe
- ○ es kostengünstig ist
- ○ ich mich gesund verhalten möchte
- ○ ich mich für digitale Technologien interessiere
- ○ ich den Informationen vertraue
- ○ ich sonst schweren Zugang zu Gesundheitsdienstleistungen habe (z.B. große Distanz oder eingeschränkte Mobilität)
- ○ ich andere Gründe habe

5 **Ich nutze <u>keine</u> digitalen Gesundheitsangebote und Anwendungen, weil…**

Interviewerhinweis: Mehrfachantworten möglich

- ○ ich Informationen auf Papier bevorzuge
- ○ ich eine persönliche Beratung bevorzuge
- ○ ich einen schlechten Internetzugang habe
- ○ ich technische Probleme habe
- ○ ich kein geeignetes Gerät besitze
- ○ ich nicht weiß, ob meine Daten sicher sind
- ○ ich den Informationen nicht vertraue
- ○ ich kein Interesse daran habe
- ○ ich andere Gründe habe

6 **Bei den folgenden Fragen geht es um digitale Gesundheitsinformationen. Ich lese ich Ihnen verschiedene Aussagen vor. Bitte wählen Sie aus den folgenden fünf Antwortmöglichkeiten: „stimme überhaupt nicht zu“, „stimme nicht zu“, „weder noch“, „stimme zu“ oder „stimme voll und ganz zu“.**

	**Stimme überhaupt nicht zu**	**Stimme nicht zu**	**Weder noch**	**Stimme zu**	**Stimme voll und ganz zu**
**6a** Ich weiß, welche Quellen für Gesundheitsinformationen im Internet verfügbar sind	○	○	○	○	○
**6b** Ich weiß, <u>wo</u> ich im Internet nützliche Gesundheitsinformationen finden kann	○	○	○	○	○
**6c** Ich weiß, <u>wie</u> ich im Internet nützliche Gesundheitsinformationen finde	○	○	○	○	○
**6d** Ich weiß, wie ich das Internet nutzen kann, um Antworten auf meine Fragen rund um das Thema Gesundheit zu bekommen	○	○	○	○	○
**6e** Ich weiß, wie ich Informationen aus dem Internet so nutzen kann, dass sie mir weiterhelfen	○	○	○	○	○
**6f** Ich bin in der Lage, Informationen, die ich im Internet finde, kritisch zu bewerten	○	○	○	○	○
**6g** Ich kann im Internet zuverlässige von fragwürdigen Informationen unterscheiden	○	○	○	○	○
**6h** Wenn ich gesundheitsbezogene Entscheidungen auf Basis von Informationen aus dem Internet treffe, fühle ich mich sicher dabei	○	○	○	○	○

Alpha-Kurzdiagnostik Lesen

Ich möchte nun erfahren, wie gut Sie lesen können. Dazu stelle ich Ihnen verschiedene Aufgaben, bei denen ich Sie bitte Silben, Wörter oder Texte laut vorzulesen.

Interviewerhinweis: Bei Teilnehmenden aus Integrations- und Alphabetisierungskursen wird die Kurzdiagnostik übersprungen. Dafür bitte hier die Kursbezeichnung eintragen:

Kursbezeichnung: ________________________

7a **Zeigen Sie auf den ersten Buchstaben des genannten Wortes und sprechen Sie ihn laut aus.** (Alpha-Level 1)

Interviewerhinweis: Sprechen Sie die Wörter, die durch die Bilder angezeigt werden, deutlich aus (z.B.Fisch). Bitten Sie die Person, den Anfangsbuchstaben des Wortes zu nennen und auf den Buchstaben zu deuten. Wörter sind: Fisch, Paket, Geld, Apfel, Käse, Butter

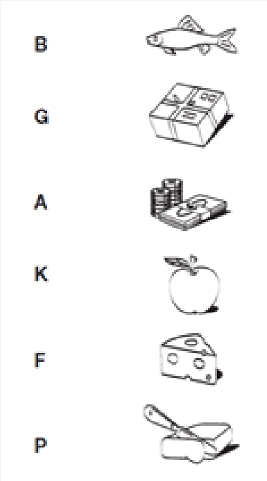

Unkorrigierte Lesefehler: _______

Interviewerhinweis: Sowohl das Aussprechen des Lautes (z.B. f) als auch die Benennung des Buchstabens (z.B. ef) werden als richtig gewertet.

7b **Lesen Sie die Silben laut vor.** (Alpha-Level 1)

Ha	Go	Mau
Ha	Ga	Mau
Hu	Ga	Wau
Hu	Go	Wau

Unkorrigierte Lesefehler: ________

Interviewerhinweis: Die Aufgabe ist vollständig erfüllt, wenn die Teilnehmenden die Silben fehlerfrei lautieren.

7c **Lesen Sie die Silben bzw. Wörter laut vor.** (Alpha-Level 1)

Pa	Oma	leben	Tomate
Mo	Mama	holen	Banane
Er	Ofen	sagen	Melone

Unkorrigierte Lesefehler: ________

Interviewerhinweis: Die Aufgabe ist vollständig erfüllt, wenn die Teilnehmenden die Silben/Wörter fehlerfrei lautieren.

7d **Lesen Sie die Wörter laut vor.** (Alpha-Level 1)

Eimer	Fisch	Schule
Pinsel	Fuchs	Ausbildung
Farbe	Hündin	Arbeit
schön	Pferd	Stelle

Unkorrigierte Lesefehler: ________

Interviewerhinweis: Die Aufgabe ist vollständig erfüllt, wenn die Teilnehmenden die Wörter fehlerfrei lautieren.

7e **Lesen Sie die Wörter laut vor.** (Alpha-Level 2)

Baumhaus<br>Hoftor	Handtuch<br>Waschbecken	Frühstück<br>Abendessen

Unkorrigierte Lesefehler: ________

Interviewerhinweis: Die Aufgabe ist vollständig erfüllt, wenn die Teilnehmenden die meisten dieser Wörter (ggf. mit Stockung und/oder teilweise lautierend) laut lesen können.

7f **Lesen Sie den Text laut vor.** (Alpha-Level 2)

Interviewerhinweis: Zeit stoppen. Kein Fehler sind Verlesungen, die eigenständig während des Lesens korrigiert werden. Fehler sind Verlesungen, Wortauslassungen, das Fehlen einer Endung.

Eva ist froh.
Sie hat einen Job bekommen!
Sie wird im Laden helfen.
Sie soll Sachen in die Regale
räumen und aufräumen.
Morgen um acht soll sie anfangen.
Sie wird jeden Tag vier Stunden arbeiten.

Aufgabe erfüllt:

○ Ja (max. 30 Sekunden, max. 2 unkorrigierte Lesefehler)

○ Nein

7g **Lesen Sie den folgenden Text laut vor. Im Anschluss werde ich Ihnen einige kurze Frage stellen.** (Alpha-Level 3)

Intervierwerhinweis: Zeit stoppen. Kein Fehler sind Verlesungen, die eigenständig während des Lesens korrigiert werden. Fehler sind Verlesungen, Wortauslassungen, das Fehlen einer Endung.

Wie man mit alten Geräten Geld verdient

Alte Elektrogeräte müssen zu Sammelstellen gebracht werden. Sie gehören nicht in den Müll. Die Geräte werden in einzelne Teile zerlegt. Diese werden dann an Firmen verkauft. Metallteile werden dann zum Beispiel geschmolzen. Kupfer, Gold und Silber sind schwer. Sie sammeln sich deshalb am Boden des Schmelzofens. Die glühende Masse kann abgelassen werden. Das flüssige Metall kühlt dann ab. Wenn es erkaltet ist, kann es wiederverwendet werden.

Wohin müssen alte Elektrogeräte gebracht werden? Zielt auf: Sammelstellen

Was geschieht mit den Metallteilen? Zielt auf: Sie werden geschmolzen

Aufgabe erfüllt:

○ Ja (max. 45 Sekunden, max. 4 unkorrigierte Lesefehler, Fragen richtig beantwortet)

○ Nein

7h **Lesen Sie den folgenden Text laut vor. Im Anschluss werde ich Ihnen einige kurze Frage stellen.** (Alpha-Level 3)

Interviewerhinweis: Zeit stoppen. Kein Fehler sind Verlesungen, die eigenständig während des Lesens korrigiert werden. Fehler sind Verlesungen, Wortauslassungen, das Fehlen einer Endung.

Pflegekräfte gesucht

Es gibt immer mehr ältere Menschen. Viele ziehen in ein Altersheim, wenn sie sich nicht mehr selbstständig versorgen können. Dort wird für sie gekocht, und ein Arzt oder eine Krankenschwester ist immer erreichbar. Man hilft ihnen dort auch bei kleinen Problemen, zum Beispiel beim Baden. Allerdings fehlen Pflegekräfte in den Altersheimen. Deshalb fördert das Arbeitsamt Umschulungen zum Altenpfleger: Wer arbeitslos ist und diese Umschulung machen will, kann anschließend in einem Altersheim Arbeit finden.

Warum ziehen ältere Menschen in ein Altersheim? Zielt auf: Sie können sich nicht mehr selbstständig versorgen

Welches Problem haben die Altersheime? Zielt auf: Die Überschrift oder auf „fehlen Pflegekräfte". Textformulierung wird nicht wörtlich aufgenommen. Hier ist globales Textverstehen, also die Gesamtaussage des – semantisch relativ einfachen – Textes gefragt.

Aufgabe erfüllt:

○ Ja (max. 45 Sekunden, max. 4 unkorrigierte Lesefehler, Fragen richtig beantwortet)

○ Nein

7i **Lesen Sie den folgenden Text zügig vor. Im Anschluss werde ich Ihnen einige kurze Frage stellen.** (Alpha-Level 4)

Interviewerhinweis: Zeit stoppen. Kein Fehler sind Verlesungen, die eigenständig während des Lesens korrigiert werden. Fehler sind Verlesungen, Wortauslassungen, das Fehlen einer Endung.

Bundesliga als Job-Motor

Frankfurt, 27.02.2016. Die Bundesliga boomt. Eine Zeitschrift für Fußball-Fans hat kürzlich vermeldet: In der vergangenen Saison haben die 18 Klubs der Bundesliga mit über 2,6 Milliarden Euro einen neuen Umsatzrekord gemacht.

Im Vergleich zum Vorjahr entspricht dies einem Plus von fast sieben Prozent. Die 2. Bundesliga setzte mit 505 Millionen Euro erstmals mehr als eine halbe Milliarde um. Damit übertraf der Umsatz beider Ligen zusammen zum ersten Mal drei Milliarden Euro.

Der Erfolg der Ligen hat eine große Wirkung auf den Arbeitsmarkt: Über 50.000 Jobs stehen im Zusammenhang mit Bundesliga-Fußball, meldet die Deutsche Fußball-Liga. Einerseits stellen die Fußballvereine nicht nur Fußballer ein. Platzwarte, Sekretärinnen, Masseure, auch Köche und Verkäuferinnen im Fan-Shop werden gebraucht. Andererseits hat die Deutsche Fußball-Liga, die die Bundesliga organisiert, selbst eine ganze Reihe von Mitarbeitern. Eine Unternehmensberatung sieht die Bundesliga sogar für 110.000 Jobs verantwortlich. Schließlich arbeiteten auch eine ganze Reihe von Event- und Marketingagenturen ausschließlich für die Bundesliga. Deren Angestellte müsse man mitzählen, so die Beratungsfirma. Um in der Bundesliga arbeiten zu können, gibt es keine optimale Ausbildung: Die meisten Angestellten seien Quereinsteiger. Sie verbindet nur eins: Fast alle hätten schon seit ihrer Kindheit sehr großes Interesse an Fußball.

Wie hoch ist der Umsatz beider Ligen? Zielt auf: über drei Milliarden Euro

Was haben viele der Angestellten gemeinsam? Sinngemäß: Sie sind Quereinsteiger und/oder haben schon seit ihrer Kindheit großes Interesse an Fußball

Aufgabe erfüllt:

○ Ja (max. 120 Sekunden, max. 10 unkorrigierte Lesefehler, Fragen richtig beantwortet)

○ Nein

Soziodemographie

Abschließend möchte ich ein paar Informationen zu Ihrer eigenen Person erfragen.

8 In welchem Jahr sind Sie geboren?

Jahr: |__|__|__|__|

9 Welchem Geschlecht ordnen Sie sich zu?

- ○ Mann
- ○ Frau
- ○ Divers

10 In welchem Land sind Sie geboren?

Interviewerhinweis: Bitte verwenden Sie die heutige Staatsbezeichnung, auch wenn das Gebiet früher einem anderen Staat zugeordnet war. Zum Beispiel „Tschechien" statt „Tschechoslowakei"

- ○ Deutschland
- ○ Anderes Land, welches? ________________________________

11 Welche Sprache sprechen Sie hauptsächlich zu Hause?

- ○ Deutsch → *Interviewerhinweis: weiter mit Frage 13*
- ○ Eine andere Sprache, welche? ___________________________

12 **Wie gut können Sie in Ihrer Herkunftssprache sprechen, lesen und schreiben? Bitte wählen Sie aus den folgenden Antwortmöglichkeiten: „Sehr schlecht", „Schlecht", „Mittel", „Gut „ oder „Sehr gut".**

	Sehr schlecht	Schlecht	Mittel	Gut	Sehr gut
**12a** Sprechen	◯	◯	◯	◯	◯
**12b** Lesen	◯	◯	◯	◯	◯
**12c** Schreiben	◯	◯	◯	◯	◯

13 **Welchen höchsten allgemeinbildenden Schulabschluss haben Sie?**

Interviewerhinweis: Ordnen Sie bitte im Ausland erworbene Abschlüsse einem gleichwertigen deutschen Abschluss zu.

- ◯ (Noch) kein Schulabschluss
- ◯ Abschluss an Hauptschule oder Realschule (nach 9. oder 10. Klasse)
- ◯ Abschluss an Gymnasium oder Fachoberschule (nach 12. oder 13. Klasse)
- ◯ Anderer Schulabschluss, und zwar: __________________________

14 **Welchen höchsten beruflichen Ausbildungsabschluss haben Sie?**

Interviewerhinweis: Ordnen Sie bitte im Ausland erworbene Abschlüsse einem gleichwertigen deutschen Abschluss zu.

- ◯ Lehre (beruflich-betriebliche Ausbildung)
- ◯ Berufsschule, Handelsschule (beruflich-schulische Ausbildung)
- ◯ Fachschule (z.B. Meister-Technikerschule, Berufs- oder Fachakademie)
- ◯ Bachelor-Abschluss an einer Universität oder Hochschule
- ◯ Diplom- oder Masterabschluss an einer Universität oder Hochschule
- ◯ (Noch) kein beruflicher Abschluss
- ◯ Anderer Abschluss, und zwar: ___________________________

15 Welcher Hauptbeschäftigung gingen Sie in den letzten 6 Monaten nach?

- ◯ Vollzeit heimische Hausarbeit/ Hausfrau oder Hausmann
- ◯ Vollzeitarbeit
- ◯ Teilzeitarbeit
- ◯ Arbeitssuchend
- ◯ Ausbildung in Vollzeit
- ◯ Erwerbsunfähig
- ◯ Etwas anderes: ____________________________

Vielen Dank für Ihre Teilnahme!

Ende des Interviews: ____:____ Uhr
