# Supplementary material for: Digital Health Literacy in Adults With Low Reading and Writing Skills Living in Germany: Mixed Methods Study
Source: JMIR Hum Factors. 2025 May 22;12:e65345. doi: 10.2196/65345 (PMC12121537; doi:10.2196/65345)
Supplement: Multimedia Appendix 2 [file humanfactors-v12-e65345-s002.pdf]

**Einverständniserklärung zur Teilnahme an der Befragung**

Liter@te – Digitale Gesundheit finden, verstehen, benutzen

- Für das BIPS -

Das Informationsschreiben habe ich erhalten und die Studie wurde mir erklärt. Mir wurde die Gelegenheit gegeben, Fragen zur Studie zu stellen. Falls ich weitere Fragen habe, weiß ich, an wen ich mich wenden kann. Die Telefonnummer 0157/36765498 ist Montag bis Freitag von 09:00 bis 15:00 Uhr für Rückfragen erreichbar. Die E-Mail Adresse ist literate@leibniz-bips.de

Ich wurde darüber informiert,

- dass meine Teilnahme an dieser Studie freiwillig ist.
- dass ich nicht alle Fragen beantworten muss.
- dass ich jederzeit und ohne Angabe von Gründen sagen kann, dass ich nicht mehr an der Studie teilnehmen möchte.
- dass mir durch die Entscheidung, nicht an der Studie teilzunehmen, keine Nachteile entstehen.
- dass meine Angaben in der Befragung anonym ausgewertet werden.
- dass meine Einverständniserklärung und meine Kontaktdaten sicher im BIPS aufbewahrt und nach Projektende (spätestens zum 30.06.2023) vernichtet werden.
- dass die anonymisierten Daten 10 Jahre nach Projektende gelöscht werden (30.06.2033).
- dass ich ein Recht auf Auskunft und Berichtigung der von mir erhobenen personenbezogenen Daten habe.
- dass ich ein Beschwerderecht bei der zuständigen Aufsichtsbehörde habe.

**Ich möchte an der Studie teilnehmen und erkläre mich mit der im Informationsschreiben beschriebenen Verarbeitung meiner Daten einverstanden.**

| | |
|---|---|
| **Nachname** | **Vorname** |
| | |
| **Datum** | **Unterschrift** |

Leibniz-Institut für Präventionsforschung und Epidemiologie – BIPS

Literate

# Einverständniserklärung zur Teilnahme an der Befragung

Liter@te – Digitale Gesundheit finden, verstehen, benutzen

- Für den Teilnehmenden -

Das Informationsschreiben habe ich erhalten und die Studie wurde mir erklärt. Mir wurde die Gelegenheit gegeben, Fragen zur Studie zu stellen. Falls ich weitere Fragen habe, weiß ich, an wen ich mich wenden kann. Die Telefonnummer 0157/36765498 ist Montag bis Freitag von 09:00 bis 15:00 Uhr für Rückfragen erreichbar. Die E-Mail Adresse ist literate@leibniz-bips.de

Ich wurde darüber informiert,

- dass meine Teilnahme an dieser Studie freiwillig ist.
- dass ich nicht alle Fragen beantworten muss.
- dass ich jederzeit und ohne Angabe von Gründen sagen kann, dass ich nicht mehr an der Studie teilnehmen möchte.
- dass mir durch die Entscheidung, nicht an der Studie teilzunehmen, keine Nachteile entstehen.
- dass meine Angaben in der Befragung anonym ausgewertet werden.
- dass meine Einverständniserklärung und meine Kontaktdaten sicher im BIPS aufbewahrt und nach Projektende (spätestens zum 30.06.2023) vernichtet werden.
- dass die anonymisierten Daten 10 Jahre nach Projektende gelöscht werden (30.06.2033).
- dass ich ein Recht auf Auskunft und Berichtigung der von mir erhobenen personenbezogenen Daten habe.
- dass ich ein Beschwerderecht bei der zuständigen Aufsichtsbehörde habe.

**Ich möchte an der Studie teilnehmen und erkläre mich mit der im Informationsschreiben beschriebenen Verarbeitung meiner Daten einverstanden.**

| | |
|---|---|
| **Nachname** | **Vorname** |
| **Datum** | **Unterschrift** |
