# Supplementary material for: Digital Health Literacy in Adults With Low Reading and Writing Skills Living in Germany: Mixed Methods Study
Source: JMIR Hum Factors. 2025 May 22;12:e65345. doi: 10.2196/65345 (PMC12121537; doi:10.2196/65345)
Supplement: Multimedia Appendix 3 [file humanfactors-v12-e65345-s003.pdf]

Appendix 2

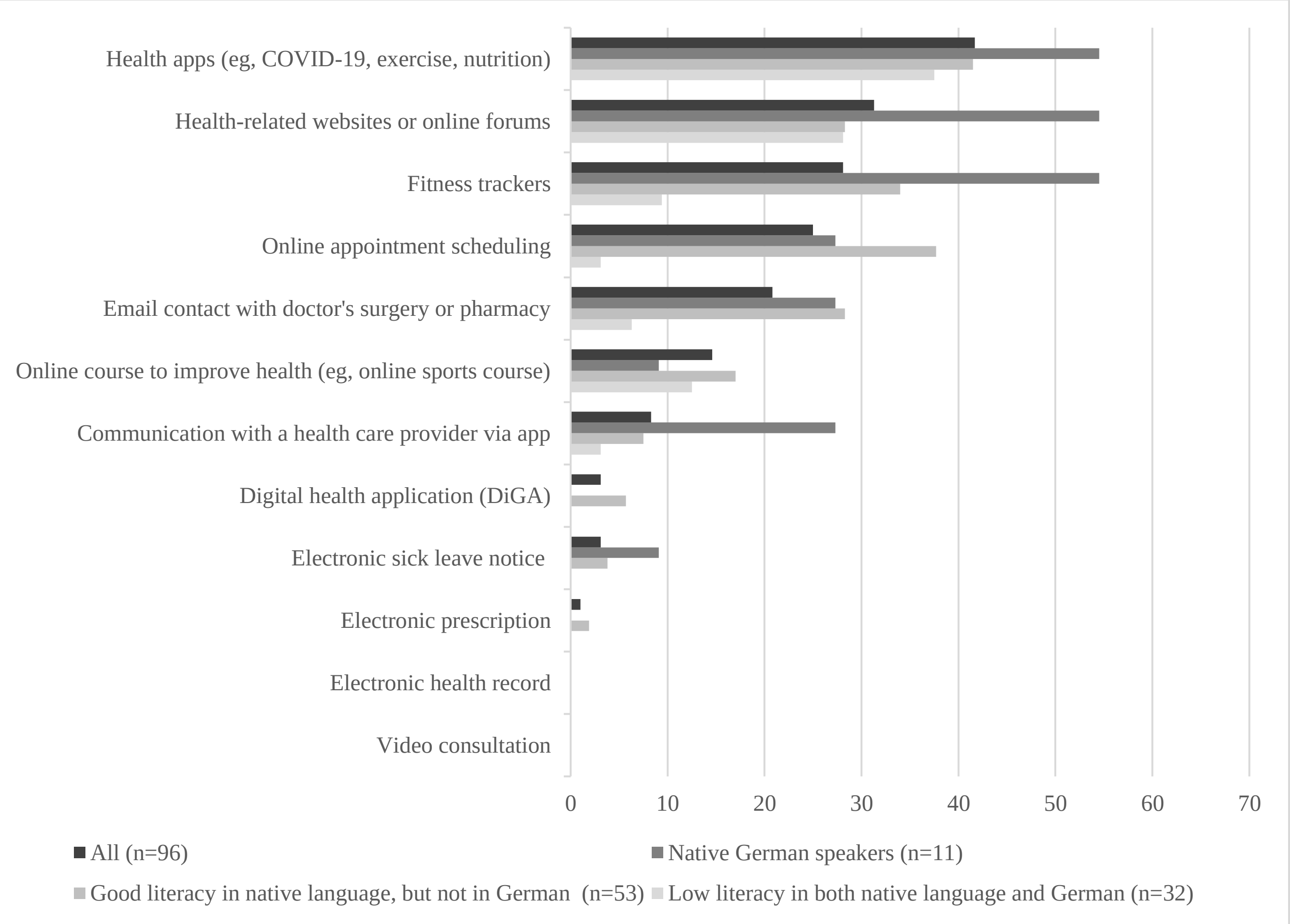

Figure S1. Percentages of adults who used different digital health technologies for health-related purposes, by language skills (multiple answers allowed)
