# Supplementary material for: Digital Health Literacy in Adults With Low Reading and Writing Skills Living in Germany: Mixed Methods Study
Source: JMIR Hum Factors. 2025 May 22;12:e65345. doi: 10.2196/65345 (PMC12121537; doi:10.2196/65345)

**COREQ (COnsolidated criteria for REporting Qualitative research) Checklist**

A checklist of items that should be included in reports of qualitative research. You must report the page number in your manuscript

where you consider each of the items listed in this checklist. If you have not included this information, either revise your manuscript

accordingly before submitting or note N/A.

**Topic**

**Item No.**

**Guide Questions/Description**

**Reported on**

**Page No.**

**Domain 1: Research team**

**and reﬂexivity**

*Personal characteristics*

Interviewer/facilitator

Credentials

Occupation

Gender

Experience and training

*Relationship with*

*participants*

1

2

3

4

5

Which author/s conducted the interview or focus group?

What were the researcher’s credentials? E.g. PhD, MD

What was their occupation at the time of the study?

Was the researcher male or female?

Authors' contri

Author informa

Aautor informa

Authors' contri

What experience or training did the researcher have?

Author informa

Relationship established

Participant knowledge of

the interviewer

6

7

Was a relationship established prior to study commencement?

What did the participants know about the researcher? e.g. personal

goals, reasons for doing the research

N/A

N/A

Interviewer characteristics

8

What characteristics were reported about the inter viewer/facilitator?

e.g. Bias, assumptions, reasons and interests in the research topic

N/A

**Domain 2: Study design**

*Theoretical framework*

Methodological orientation

and Theory

9

What methodological orientation was stated to underpin the study? e.g.

grounded theory, discourse analysis, ethnography, phenomenology,

content analysis

Data analysis

*Participant selection*

Sampling

10

11

How were participants selected? e.g. purposive, convenience,

consecutive, snowball

How were participants approached? e.g. face-to-face, telephone, mail,

email

Procedure

Procedure

Method of approach

Sample size

Non-participation

*Setting*

12

13

How many participants were in the study?

How many people refused to participate or dropped out? Reasons?

Focus groups

N/A

Setting of data collection

Presence of non-

participants

14

15

Where was the data collected? e.g. home, clinic, workplace

Was anyone else present besides the participants and researchers?

Focus group gu

N/A

Description of sample

16

17

What are the important characteristics of the sample? e.g. demographic

data, date

Table 1, Focus g

Focus group gu

*Data collection*

Interview guide

Were questions, prompts, guides provided by the authors? Was it pilot

tested?

Repeat interviews

Audio/visual recording

Field notes

Duration

Data saturation

Transcripts returned

18

19

20

21

22

23

Were repeat inter views carried out? If yes, how many?

Did the research use audio or visual recording to collect the data?

N/A

Focus group gu

Were ﬁeld notes made during and/or after the inter view or focus group? N/A

What was the duration of the inter views or focus group?

Was data saturation discussed?

Were transcripts returned to participants for comment and/or

Procedure

N/A

N/A


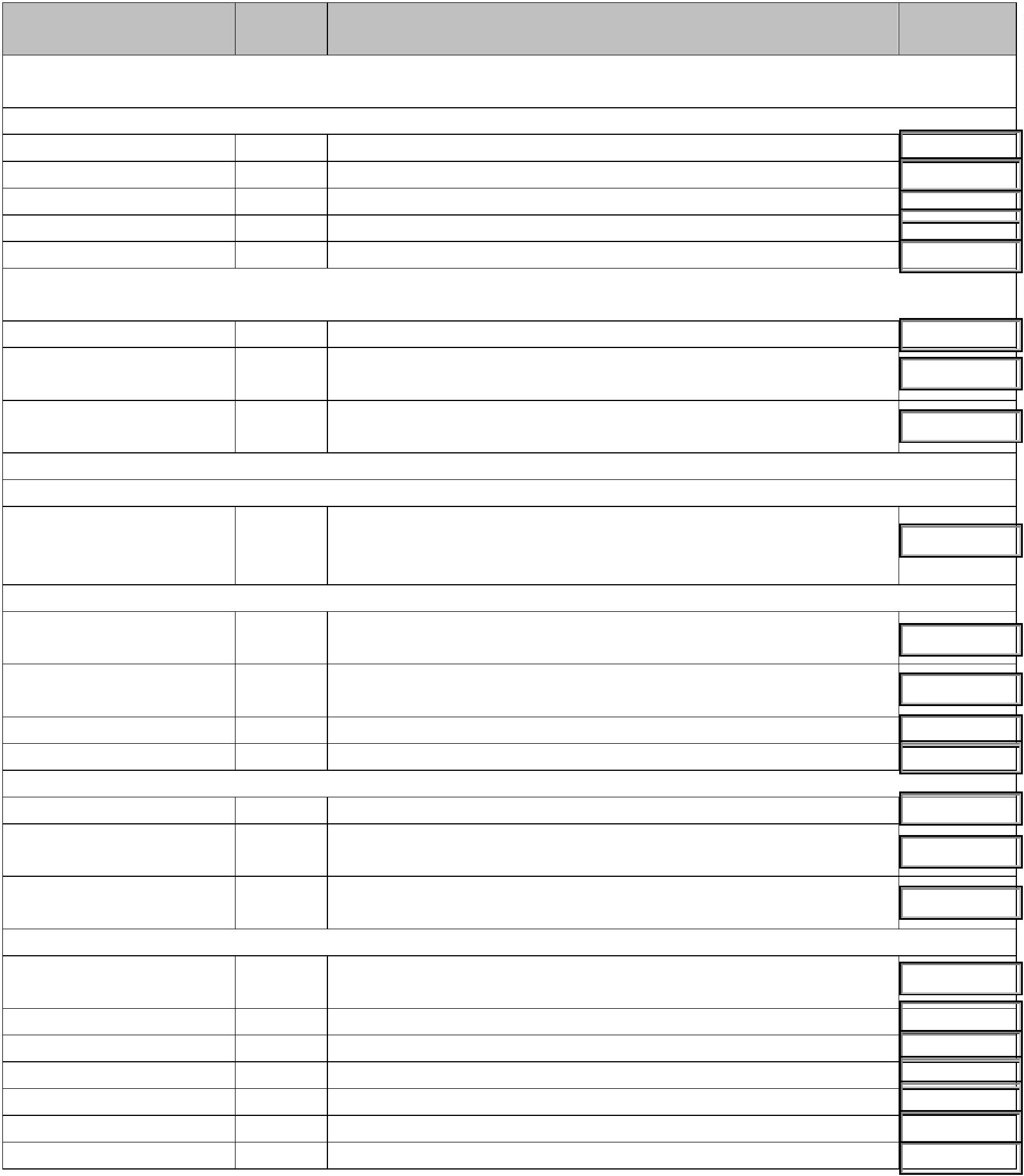

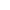


**Topic**

**Item No.**

**Guide Questions/Description**

**Reported on**

**Page No.**

correction?

**Domain 3: analysis and**

**ﬁndings**

*Data analysis*

Number of data coders

Description of the coding

tree

24

25

How many data coders coded the data?

Did authors provide a description of the coding tree?

Data analysis

N/A

Derivation of themes

Software

Participant checking

*Reporting*

26

27

28

Were themes identiﬁed in advance or derived from the data?

What software, if applicable, was used to manage the data?

Did participants provide feedback on the ﬁndings?

Data analysis

Data analysis

N/A

Quotations presented

29

Were participant quotations presented to illustrate the themes/ﬁndings?

Was each quotation identiﬁed? e.g. participant number

Table 4

Data and ﬁndings consistent

Clarity of major themes

Clarity of minor themes

30

31

32

Was there consistency between the data presented and the ﬁndings?

Were major themes clearly presented in the ﬁndings?

Is there a description of diverse cases or discussion of minor themes?

Focus groups

Focus groups

N/A

Developed from: Tong A, Sainsbury P, Craig J. Consolidated criteria for reporting qualitative research (COREQ): a 32-item checklist

for interviews and focus groups. *International Journal for Quality in Health Care*. 2007. Volume 19, Number 6: pp. 349 – 357

**Once you have completed this checklist, please save a copy and upload it as part of your submission. DO NOT include this**

**checklist as part of the main manuscript document. It must be uploaded as a separate file.**


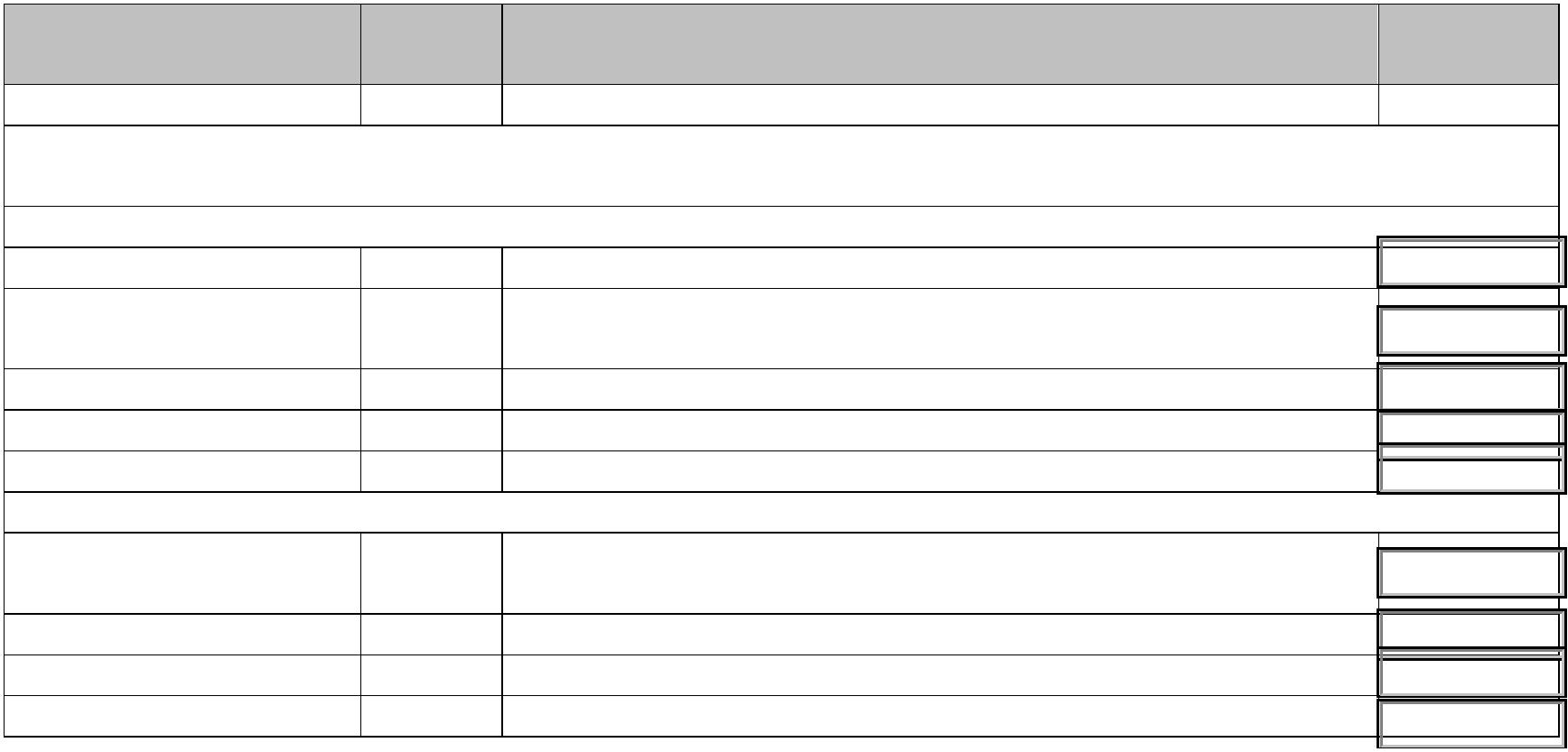

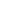

Supplement: Checklist 2 [file humanfactors-v12-e65345-s005.docx]
